# Atomic imaging of air- and electron-beam-sensitive materials by inert-gas-protected cryogenic aberration-corrected TEM

**DOI:** 10.1093/nsr/nwag225

**Published:** 2026-05-05

**Authors:** Ute Kaiser

**Affiliations:** Institute for Quantum Optics, Ulm University, Germany

Many emerging materials—such as hybrid perovskites, low-dimensional halides and organic–inorganic frameworks—are extremely fragile. They may rapidly degrade upon exposure to oxygen or moisture and often suffer severe structural damage under electron irradiation. This combination of air and electron-beam sensitivity has long limited the ability of transmission electron microscopy (TEM) to probe their intrinsic atomic structures.

In a recent study, Gang Wang, Junhao Lin and co-workers present an integrated strategy that addresses these challenges by combining inert-gas protection with aberration-corrected cryogenic TEM (cryo-TEM) [1]. Their work demonstrates how a carefully designed workflow enables atomic-resolution characterization of materials previously inaccessible to direct TEM observation.

A central element of the approach is a glovebox-integrated preparation and transfer system that maintains samples under inert conditions throughout the experiment. Specimens are prepared inside a nitrogen-filled glovebox and transferred to the microscope through a continuous liquid-nitrogen cold chain, preventing exposure to ambient atmosphere. Once inside the spherical aberration (Cs)-corrected cryo-TEM, the sample remains under cryogenic and high-vacuum conditions, preserving its intrinsic structure during imaging [1].

This work also realizes a vision Junhao Lin has advocated for many years: the establishment of reliable inert-atmosphere workflows for studying fragile materials. Turning this concept into a functioning experimental platform represents both a conceptual advance and a significant technical achievement.

The authors also address the fundamental challenge of radiation damage in high-resolution electron microscopy (EM). Beam-sensitive materials tolerate only extremely low electron doses, typically producing images with poor signal-to-noise ratios. Lin and colleagues overcome this limitation by combining low-dose imaging with dose-fractionated acquisition and computational reconstruction. Motion-correction algorithms align sequential frames to compensate for beam-induced drift, while a 3D Wiener-based filtering approach suppresses stochastic shot noise and recovers genuine structural information. Structural details can thus be extracted while keeping the accumulated electron dose below the damage threshold of the material [1].

The methodology is demonstrated for two representative systems: the highly air sensitive hybrid perovskite (FB)_2_MnCl_4_ and the 1D ferroelectric halide NbOI_3_. Conventional transfer methods lead to rapid oxidation and degradation, whereas the integrated inert-gas and cryogenic workflow preserves the intrinsic lattice structure. Using optimized low-dose conditions, the authors obtain atomic-resolution cryo-TEM images with an information limit of approximately 1.6 Å at a total accumulated dose of only about 3 e^−^ Å^−2^ [1].

More broadly, this work highlights how integrating environmental control, cryogenic instrumentation, aberration-corrected optics and computational image processing expands the capabilities of EM for materials research. Cryogenic EM (cryo-EM) has already revolutionized structural biology [2] and is increasingly applied to sensitive materials and quantum systems [3]. Earlier studies demonstrated that low-dose strategies can reveal atomic structures in beam-sensitive crystals [4,5], while cryo-EM has enabled investigations of highly reactive battery materials and interfaces [6].

During a recent visit to the authors’ laboratory, it became clear how strongly this vision has shaped the group’s research strategy. Junhao Lin’s early recognition that reliable studies of such fragile materials require workflows carried out entirely under inert conditions—practically achievable only inside a glovebox—guided the development of the experimental platform from the outset. The demanding preparation and transfer procedures rely not only on advanced instrumentation but also on the practical skills required for precise manipulation in the confined glovebox environment. These experimental capabilities lie at the heart of the project, just as much as the underlying theoretical insights; without them, the successful realization of this approach would not have been possible.

As new classes of quantum, energy and hybrid materials continue to emerge, many of which are highly sensitive to both environmental exposure and electron irradiation, the ability to visualize their atomic structures will become increasingly important. The approach demonstrated here represents an important step toward making atomic-resolution cryo-EM a practical tool for exploring fragile materials that have until now remained beyond the reach of conventional TEM.
